# The Portrait of Dr. Adolph Lippe

**Published:** 1892-05

**Authors:** C. Carleton Smith

**Affiliations:** 875 North Twentieth Street, Philadelphia


### THE PORTRAIT OF DR. ADOLPH LIPPE.

875 North Twentieth Street,
Philadelphia, April 4th, 1892.

Editor of The Homœopathic Physician:

I feel as if I could not thank you sufficiently for the most admirable likeness of our departed friend and colleague, Dr. Lippe, which you have given to the subscribers of your journal, as a most fitting memorial to the honor of that great man.

As his counterpart, it is *absolutely perfect*, being truthful even to the smallest detail, and so striking, that when I turned to this page of the journal I was actually startled. Surely, you deserve the warmest praise from every true Hahnemannian, who, through your enterprise and great kindness, will come into the possession of this unapproachable work of the photographer's art.

It is the only perfect likeness I have ever seen of the Doctor, and I would not take for it the price of many years' subscription to THE HOMŒOPATHIC PHYSICIAN. In short, I would not part with it on *any terms*.

Being a close personal friend of Dr. Lippe's, many a time have I made, when in his presence, a critical mental study of his face. Its characteristic feature was the mouth, and it stamped the man. The picture before us is, in this respect, without a single flaw. The life-like expression existing in every line drawn by the artist, Nature, may well cause us to ask if "Old Sol" was not truly inspired, when, with a few touches of his pencil of light he has given back to us the face of our beloved friend and colleague, just as I remembered it in by-gone days.

Fraternally, yours, in the cause,

C. CARLETON SMITH.

